# The Hegemony of far-Right Populism, Project 2025, and the Dangers Ahead for Science and Public Health

**DOI:** 10.1177/27551938251367853

**Published:** 2025-08-17

**Authors:** Corrado Piroddi, Lynda Gilby, Meri Koivusalo, Alison McCallum, Abbe Brown, Chloe Stephenson

**Affiliations:** 1 Postdoctoral Research Fellow, Faculty of Social Sciences, Tampere University, Kalevantie 4, 33100 Tampere, Finland; 2 Doctoral Researcher, Faculty of Health Sciences, Tampere University, Kalevantie 4, 33100 Tampere, Finland; 3 Professor of Global Health, WHO Collaboration Centre on Health in All Policies and Social Determinants of health, 528748 Tampere University, Kalevantie 4, 33100 Tampere, Finland; 4 Usher Institute, University of Edinburgh, Old College, South Bridge, Edinburgh EH8 9YL, UK; 5 Professor of Intellectual Property, School of Law, 1019 University of Aberdeen, King's College, Aberdeen AB24 3FX, UK; 6 Doctoral Researcher, Faculty of Health Sciences, Tampere University, Kalevantie 4, 33100 Tampere, Finland

**Keywords:** misinformation, disinformation, public health, project 2025, politization of health

## Abstract

According to the World Health Organization, the spread of misinformation and disinformation are dangerous threats to public health. The popular legitimacy of far-right politics in the United States, across Europe, and other continents constitutes a new phase that threatens to jeopardize countermeasures adopted by social, political, and scientific institutions to counter the phenomena of mis- and disinformation.

According to the World Health Organization, the spread of misinformation (incorrect information without the intent to mislead) and disinformation (incorrect information disseminated with malicious intent) are dangerous threats to public health.^1^ Disinformation is designed to sow discord and distrust, frequently toward civil servants, the scientific community, and public health agencies, often for personal, financial, or political gain. Disinformation can be used to blame and oppress minorities and marginalized groups, creating turmoil in emergencies, deepening societal polarization and political conflicts, undermining scientific institutions and public health intervention.^2^ The popular legitimacy of far-right politics in the United States, across Europe, and other continents symbolizes a new phase that threatens to jeopardize countermeasures adopted by social, political, and scientific institutions to counter the phenomena of mis- and disinformation.^3–5^ In this instance, public health itself becomes a target. And, in addition to false persecution and harm,^4^ dangers include the criminalization of legitimate scientific critique and evidence-based discussions by populist—and authoritarian—actors.

## The New Threat to Scientific Freedom and Public Health

Project 2025, a policy roadmap developed by the Heritage Foundation—one of the most influential U.S.-based conservative think tanks—has elicited widespread calls for caution, particularly related to harm arising from extreme versions of policies.^5,6^ With the 2024 electoral victory of Donald Trump for a second presidential term, the policy space in relation to public health and the environment is alight with speculations regarding the future stance of U.S. government action in these areas.^7^

In the history of public health, this event is not new. Political forces have challenged public health policies and scientific consensus based on questions of public interest. During the nineteenth century in Great Britain, for instance, the theory of contagion, sanitary cordons, quarantine, and vaccination campaigns was politically contested by liberal reformers in the name of freedom of movement and free trade.^8^ In the same century, contagionism found the fierce social and political opposition of merchants and industrialists; it was perceived as an illegitimate interference of states and national bureaucracies in their freedom and business.^9^

In this regard, the Trump administration's cuts to Centers for Disease Control and Prevention (CDC^10^; and federal jobs do not constitute a unique case in the United States and European nations’ political and medical history. Also, in Europe, right-wing groups and parties tend either to promote targeted welfare chauvinism,^11^ which is generally exclusionary toward ethnic, cultural, and gender minorities, or to assume a liberal orientation in the economy, supporting the commercialization of health services and goods and less statism.^12^

Project 2025 constitutes a handbook for promoting a new, conservative revolution in the United States under the next republican administration. Among its plans for administrative and legislative overhauls, the proposal to dismantle efforts to combat mis- and disinformation should concern the public. The document states repeatedly that concepts like “misinformation” and “disinformation” are repressive tools that limit freedom of speech and violate the First Amendment.^a^

It also states that the Intelligence Community (IC) “should be prohibited from monitoring so-called domestic disinformation. Such activity can easily slip into suppressing an opposition party's speech, is corrosive of First Amendment protections, and raises questions about impartiality when the IC chooses not to act”.^13^

Since Donald Trump's reelection, a video he made in 2022 and listed on his campaign website^14^ was shared by Elon Musk on X. Here, Trump stated plans to crack down on action on mis- and disinformation, similar to that outlined in Project 2025.

Undoubtedly, in criticizing the IC, Project 2025 expresses a sentiment that is broadly felt across the political spectrum. However, while in the past criticism against the U.S. intelligence agencies aimed at protecting individual and civil rights (for example, after 9/11 and the introduction of the Patriot Act), the current Trump administration seems to employ this kind of criticism in a novel way. It weaponizes such concerns to target research groups and institutions involved in research concerning public health policies and the spread of mis- and disinformation.

In the first months in office, the Trump administration not only threated to limit this kind of activity, but has taken active steps to prohibit research in these areas.^15^ For example, the Department of Defense has cut research on disinformation, violent extremism, and climate change.^16^ The Stanford Internet Observatory, which conducted high-profile work on election-related misinformation, was wound down after being targeted by lawsuits and Congressional subpoenas from republicans.^17^ And, the National Institute of Health has terminated 800 research projects on topics regarding vaccine hesitancy, health of transgender people, HIV, and misinformation.^18^

This raises the question of the dangers posed to public health scholars and other researchers with responsibility for protecting population health, well-being, and rights based on scientific evidence and application of the precautionary principle.^19^ Right-wing populism tends to prioritize executive power over considerations concerning the pluralism of voices in the public space, democratic discussions, and the balance of powers, which can limit the independence of public health professionals and potentially censor experts, public servants, and members of the scientific community.

In Hungary, for instance, Viktor Orban introduced an electoral mechanism that guarantees his party, Fidesz, a long-term supermajority and a constitutional reform that reinforced and extended Fidesz's control over the Constitutional Court of Hungary beyond the four-year parliamentary cycle.^20^ This democratic backslide had negative consequences both on academic freedom^21,22^ and population health, as Orban's welfare reforms negatively impacted labor market security, wealth, and income (despite a recentralization of health care services in the past decade.^23^ Unsurprisingly, during the COVID-19 pandemic, the populist Hungarian government ended up using discursive governance instruments to conceal negative health policy outcomes. It was therefore possible to arbitrarily manipulate Hungarian pandemic management through the centralized information control mechanism created by Orban and Fidesz during their almost fifteen years of power.^24^

Keeping in mind the Hungarian case, labelling the work on countering mis- and disinformation as political acts that violate freedom of speech is dangerous. It could undermine, if not criminalize, dissemination of and access to scientific information, and efforts to promote scientific literacy, if it contradicts the government's position. If debunking false information concerning climate change or vaccine effectiveness, for example, becomes a felony, then there is no space in the public sphere for a licit critique based on sound theory and scientific evidence. Clearly, this could at first sound like a hyperbolic statement, or a slippery-slope fallacy. Nevertheless, it should also be considered that on Jay Bhattacharya's first day as the National Institutes of Health (NIH) director, the agency directed staff to identify contracts related to fighting misinformation or disinformation. The NIH's official email defined this request as an attempt to address any contract that could be used to censor Americans.^25^

In addition, eroding trust in the scientific community risks undermining the dialogue between communities and public health professionals. This interaction between government, health care providers, and social groups with different health needs and priorities is fundamental to designing health policies that are inclusive, sustainable, and culturally appropriate.^26^

## Undermining Democratic Values and Weaponizing Health Policies

In considering the relationship between populism and public health, discussion of two other phenomena is essential: over-politicization, and the reduction of the truth–value relationship of scientific and technical facts to political positioning.

In the context of growing nationalism, over-politicization of public health could be seen as an attempt to produce an extreme form of societal and political polarization, which enables the centralization of executive power at the expense of elected legislature and judicial systems and thereby limits the influence of balancing powers in the public sphere. This global tendency threatens the stability of so-called rules-based democracies in Europe and North America, the independence and impartiality of scientific research, policy, and public health advocacy.

Right-wing populist groups and parties take advantage of people's fears and global emergencies.^27^ While publicly affirming access to health care for their citizens, they are likely to deprive the scientific community of the freedom of research and critique and limit eligibility for funding. Technical aspects of public health policies have thus become objects of political conflicts, with positions amplified that seek to undermine the partnerships between populations and public health and the role of public health professionals as honest brokers and a trusted, expert voice.^28^

It also denies people access to evidence and evidence-based prevention and treatment, as well as weakening scientific literacy. While public health professionals cannot impose policy choices on governments, they can criticize them by providing scientific evidence and analysis of impacts. Through public communication, they can provide evidence-based critique and assessments, and correct political biases and incorrect information as it emerges. Over-politicizing the public sphere can also make social minorities voiceless and amplify anti-health antagonists. For public health professionals, an environment of regulatory and policy chill may slip into coercion against designing policies that prioritize protection of population health and addressing inequalities where these ideas contradict government positions.

Since the 1980s, mainstream politics in Europe and the United States have tended to favor science, technical, and financial expertise. Advocates of technocracy^29^ assumed that political questions could be addressed through application of scientific expertise, aligned to economic policies focused on economic growth, liberalization of markets, and reduction of public debt. While the effectiveness of this approach on health is subject to significant critique, reasoned debate was possible.

With the reelection of Donald Trump, pseudo protectionism and neomercantilism are likely to be prioritized by global and regional powers and transnational actors. In this new conjuncture, security, key interests, and power of the state and its executive branch are now prioritized over the well-being of citizens, the welfare of the population, and public scientific discussion.

The weaponization of vaccine production, hoarding, and disinformation campaigns witnessed during the pandemic, a situation usually only seen during periods of acute conflict and war, is likely to be replicated by creation of new controversies and marginalization of those affected.^30,31^

## Conclusion: From Technocratic Health Policies to Over-Politicization of Health

In this emergent phase, where power politics is becoming prominent, the risk to the public space is no longer the primacy of technocratic power over democracy and collective rights. The risk to the public sphere in current democracies is to fall victim to an aggressive process of over-politicization that contains antidemocratic, authoritarian, and anti-scientific traits. The attack against the critical role of science and experts in public debate, and the stigma against those who openly contrast the nefarious effects of disinformation, are alarming signs of the increasing weight of such a risk.

## Notes

a. The First Amendment protects freedom of speech, giving everyone the right to hear different ideas regarding every issue and to formulate publicly their own judgments about those questions without government interference or limitations. Limits to the Amendment concern obscenity, defamatory speech, false advertising, threats, and incitement to lawless action.
